# Efficacy of Agricultural Residue-Derived Biochar for Tackling Cadmium Contamination in an Aqueous Solution

**DOI:** 10.3390/molecules29153545

**Published:** 2024-07-27

**Authors:** Qinghai Liu, Zhengguo Song, Jingwen Li, Chongshuang Pan, Weiwen Qiu

**Affiliations:** 1 Institute of Agricultural Quality Standard and Testing, Tibet Academy of Agricultural and Animal Husbandry Sciences, Lhasa 850032, China; liuqinghai1985@gmail.com (Q.L.); panchongshuang@126.com (C.P.); 2 Agricultural and Livestock Products Engineering Technology Research Center of Tibet Autonomous Region, Lhasa 850032, China; 3 Department of Civil and Environmental Engineering, Shantou University, Shantou 515063, China; forestman1218@163.com (Z.S.); 19jwli1@stu.edu.cn (J.L.); 4 The New Zealand Institute for Plant and Food Research Limited, Private Bag 3230, Hamilton 3240, New Zealand

**Keywords:** agricultural residue derived-biochar, Cd(II), adsorption capacity

## Abstract

This study aimed to investigate the efficacy of biochar, produced from different agricultural residues varying in lignin and cellulose content and subjected to different pyrolysis temperatures, in removing cadmium ions (Cd (II)) from an aqueous solution. This removal process is crucial for protecting human health and the environment. Specifically, the study focused on the adsorption behaviors of Cd (II) by the biochars made from rice husk biochar (RHB), maize straw biochar (MSB), peanut shell biochar (PSB), cottonseed shell biochar (CHB), and mulberry leaf biochar (MLB), which were prepared at 300 °C and 600 °C. The results indicated that the type of agricultural residue used to produce biochar significantly influenced the adsorption of Cd (II). Notably, mulberry leaf biochar prepared at 300 °C (MLB-300) demonstrated the highest adsorption efficiency, achieving a maximum adsorption capacity of 42.2 mg g^−1^. Batch adsorption experiments assessed the impact of various factors, including system pH, NO_3_^−^ concentration, and adsorption duration. The adsorption kinetics were better described by the pseudo-second-order model than the pseudo-first-order model. Moreover, the study found that the lignin content of the biochar plays a major role in determining the adsorption capacity. The surface characteristics of biochar, influenced by the types of agricultural residues and preparation temperature, directly impact its adsorption mechanism and capacity. While biochar produced at 300 °C showed optimal Cd(II) adsorption, those processed at 600 °C were less effective, likely due to the loss of functional groups at higher temperatures.

## 1. Introduction

Over recent years, anthropogenic inorganic pollutants emitted into the environment have increased in parallel with industrial and urbanization development. Toxic heavy metals, such as cadmium (Cd), copper (Cu), arsenic (As), lead (Pb), and zinc (Zn), are derived from mining, metal manufacturing, and agricultural chemical use, posing a significant threat to the wellbeing of human and animal species and plant productivity. Cd is commonly used in many industrial products as a main agent or additive/stabilizer. The untreated or accidental release of Cd-containing wastewater into the environment is responsible for its toxicity to soil and aquatic systems. Additionally, the application of phosphate-based fertilizer and pesticides on agricultural land could also contribute to Cd pollution. Due to its peculiar characteristics, e.g., equal ionic radius, same valence state/positive charges with calcium (Ca), and lower stability in soils, Cd is more easily mobilized than other toxic heavy metals, and it can be taken up by edible parts of plants, eventually making its way into the human food chain [1,2,3]. The World Health Organization [4] recommends that the Cd concentration in drinking water be below 3 µg/L. Long-term exposure to Cd can lead to biological dysfunction in the kidneys, heart, liver, salivary glands, prostate, spleen, testes, epididymis, and thymus [5].

To reduce Cd contamination in wastewater prior to discharge, several methods, including chemical precipitation or membrane filtration, ion-exchange, activated carbon, and various adsorption and oxidation processes, have been applied to Cd remediation [6,7]. Among them, the adsorption method has gained widespread use for removing heavy metals from the environment due to its simplicity, versatility, and cost-effectiveness in recent years. Various types of adsorbents, such as biochar, graphene, activated carbon, and carbon nanotubes, have been developed [7]. In these adsorbents, biochar has garnered significant attention for its affordability, porous and loosely structured composition, and abundance of reactive carboxyl and hydroxyl groups on their surfaces, making it an effective solution for various environmental challenges. Biochar is a carbon-rich material with a fine texture that is produced mainly from organic substances such as algae, manure, agricultural residues, energy crops, activated sludge, and forest residues using the pyrolysis process under oxygen-limited conditions at appropriate temperatures [8]. Biochar applied to soils is a general agricultural practice to improve soil fertility, especially beneficial to soils with low organic matter content. Such additions can also serve as bio-adsorbents to remediate inorganic/organic pollutants-contaminated soil or water environments by reducing pollutants mobility. Agricultural residues, specifically plant residues, are extensively employed for producing biochar due to their rich carbon content, abundant availability in nature, and potential environmental benefits. Typically, agricultural residues (plant residues) consist of two main phases, i.e., the inorganic phase and the organic phase. The inorganic phase comprises components of silicate, sulfate, hydroxide, or other inorganic salts. On the other hand, the organic phase mainly consists of three important components: lignin, cellulose, and hemicellulose. Several factors, such as grain size, temperature, thermal rate, and residence time, affect the quality and chemical composition of biochar during the pyrolysis process. Some studies have explored the use of lignocellulosic materials as cheaper bio-adsorbents. Agricultural restudies, such as onion skins [9,10], peanut skin [11], walnut shells [12], and maize cobs [13], have been investigated for this purpose. Some of these agricultural residue-derived biochars can be further modified to improve their adsorbent behavior, stability, and/or adsorption capacity. Such modifications can be low-cost and simple, making them highly desirable for large-scale treatment of industrial and mining wastewater prior to discharge. The characteristics of biochars vary significantly based on the type of feedstock and production conditions [14]. During the pyrolysis of biomass, cellulose and lignin, the main compounds, produce volatile products with different structures and properties, which leads to variations in the types, quantities, and characteristics of the pyrolysis products. Experiments conducted by Kawamoto et al. [15] have shown that the fracture of ether bonds is the main process during the pyrolysis of lignin. Cellulose retains its crystalline and highly ordered structure at temperatures below 250 °C. However, at temperatures of 250 °C or higher, cellulose undergoes a transformation, resulting in the formation of a new polymer with a furan framework, a hydroxyl framework, unsaturated hydrocarbon chains, and a notable presence of carboxyl and carbonyl groups. As the temperature continues to rise, cellulose experiences further dehydration and disproportionation, especially at temperatures exceeding 310 °C. This process leads to the emission of CO and CO_2_ and the formation of highly condensed aromatic polymers. Chua et al. [16] conducted a study and found that hemicellulose decomposes rapidly at temperatures below 220 °C, while cellulose and lignin decompose in the ranges of 140–360 °C and 160–900 °C, respectively, under non-catalytic conditions. Both lignin and cellulose are abundant in various functional groups, and FTIR studies have shown that hemicellulose, cellulose, and lignin have different oxygen-containing functional groups, including olefin, ester, aromatic hydrocarbon, ketone, and alcohol. Infrared absorption spectra of OH and C-O in cellulose are the highest, while hemicellulose has a higher C=O content. Lignin has complex infrared absorption spectra, indicating that it may contain Methoxyl-O-CH_3_, C-O-C vibration, and C=C vibration stretch-aromatic ring compounds.

The crop residues/agricultural by-products investigated for their Cd removal capacity in this study were rice husk, maize straw, peanut shell, cottonseed shell, and mulberry leaf. These materials are derived from agricultural activities and consist mainly of cellulose, hemicellulose, and lignin. Meanwhile, they also comprise a low to moderate amount of ash, silica, fatty acids, and phenolic compounds. The organic and inorganic compositions of these five plant residues may vary, making them suitable feedstocks for various applications, such as producing effective bio-absorbents or biofuels. Instead of discarding them, these residues can be reused or pretreated in environmentally responsible ways to extract their full potential. In this study, biochar produced from five types of plant residue biomass with different lignin-cellulose ratios was compared for its adsorption capacity for the heavy metal Cd(II) under the same conditions. The study also investigated the adsorption kinetics and isotherms, the effects of pH and ionic strength on adsorption, and evaluated the morphological structure and component changes of plant residues derived from biochar before and after adsorption to explore the Cd(II) adsorption mechanism of biochar and the relationship between lignin-cellulose content and adsorption capacity.

## 2. Results and Discussion

### 2.1. Characterization of Biochar

#### 2.1.1. Agricultural Residues Composition

The lignocellulosic biomass mentioned in Table S1 exhibits varying biochemical compositions with regards to lignin and cellulose content. Among them, cotton seed shell hair has the highest cellulose content (70%), which is 2.8 times higher than that of mulberry leaf (18.9%), which has the lowest cellulose content. In contrast, peanut shell biomass contained the most significant amount of lignin (34.7%), followed by mulberry leaf (32.8%), rice husk (24.6%), and approximately 14% in corn stover and cotton seed shell hair. In terms of the lignin-to-cellulose ratio, mulberry leaf had twice as much lignin as cellulose (2:1), while rice husk, cotton seed shell hair, and corn stover possessed 2–5 times more cellulose compared to lignin. Only peanut shells showed an equivalent lignin and cellulose content. These variations in biochemical composition in agricultural residues are a result of a combination of genetic factors and environmental conditions.

In general, woody plant residues such as mulberry tree leaves have a high lignin content and a relatively high protein content, but a lower cellulose content. Conversely, residues from cereal crops (rice and maize) and fiber crops (cotton) tend to have a higher cellulose content. Peanut shells, in particular, have a lower ash content, primarily composed of inorganic minerals and silica, compared to other residues (Table 1).

#### 2.1.2. SEM Analysis

**Figure 1:**
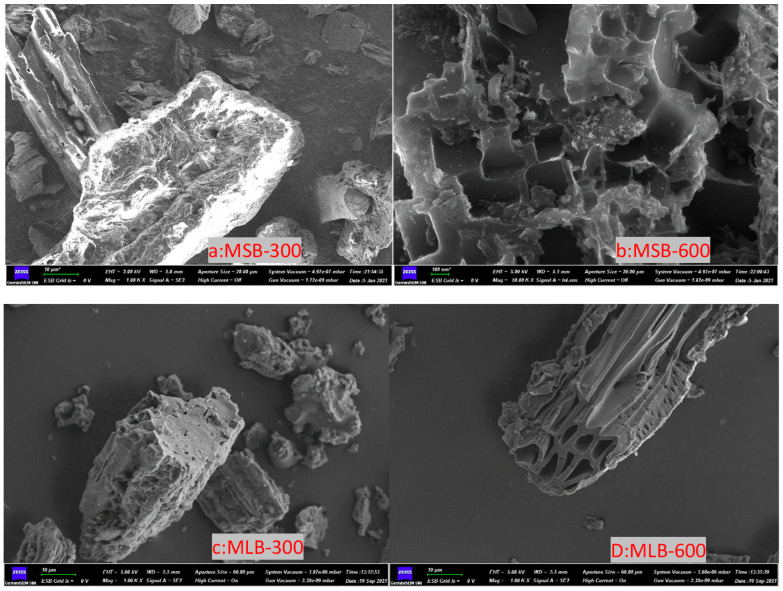
SEM observation of maize straw and mulberry leaf biochar (300 and 600 °C).

SEM characterization was applied to analyze the surface morphology of the studied biochar. It can be seen from Figure 1 that the two biochars (maize straw- and mulberry leaf-derived) generated at 300 °C still retained the original skeleton and structure of the raw materials [17]. With the increase in pyrolysis temperature (600 °C), the surface of biochar becomes rougher, and the pore structure becomes more obvious [18]. 

#### 2.1.3. Elemental Analysis and BET Surface Area

The biochar prepared from rice husk, maize straw, peanut shell, and cottonseed hull increased the carbon content and decreased the hydrogen content and oxygen content with the increase in temperature (Table 1). For MLB, the carbon content did not change significantly with the increase in temperature, but the oxygen content increased. The content of the N element in MLB was higher than that of other biochar because the protein content in MLB leaves was high [19], and N was difficult to remove in the pyrolysis process. The ratio of H/C and (O + N)/C in biochar was used to judge the aromaticity and polarity of biochar, respectively. The smaller the H/C was, the higher the aromaticity was; the larger the (O + N)/C was, the greater the polarity was. For MSB-300, H/C and (O + N)/C are 0.068 and 0.546, respectively. For MSB-600, H/C and (O + N)/C are 0.028 and 0.384, respectively. The H/C and (O + N)/C of biochar prepared at a pyrolysis temperature of 600 °C both decreased, indicating that the aliphatic properties of biochar gradually weakened, but the aromatics became higher and the polarity became smaller.

As shown in Table 1, the specific surface area of RHB, MSB, PSB, and CHB gradually increased with the rise in preparation temperature. For instance, the specific surface area of cottonseed shell biochar increased from 1.59 m^2^·g^−1^ at 300 °C to 292.31 m^2^·g^−1^ at 600 °C. The total pore volume of biochar dictates the available space for adsorbate molecules. A higher pore volume permits the retention of more Cd^2^⁺ ions. Furthermore, well-developed mesopores and macropores improve the accessibility of micropores, allowing Cd^2^⁺ ions to reach the adsorption sites with greater ease. In this study, a similar trend was observed, where higher temperatures resulted in an increased total pore volume of biochar. This can be attributed to the opening and enlargement of mesopores and micropores in biochar under high-temperature conditions, thereby increasing the overall pore volume. However, the specific surface area and total pore volume of MLB-derived biochar remained relatively consistent between 300 °C and 600 °C.

#### 2.1.4. FTIR Analysis

**Figure 2:**
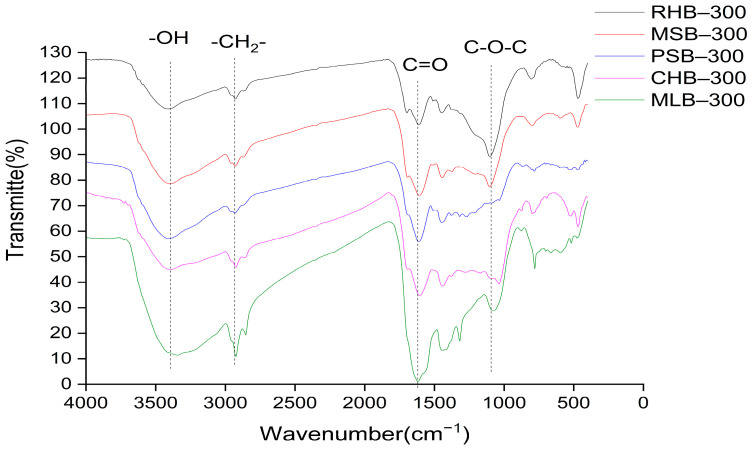
FTIR spectra of different biochars after being heated at 300 °C.

The presence of functional groups plays a crucial role in adsorption processes. Research conducted by Ahmed, Zhou, Ngo, and Guo [20] revealed that biochar containing a higher proportion of oxygen-containing functional groups exhibited stronger cation exchange capacity and enhanced adsorption capabilities. Figure 2 presents the FTIR spectra of five biochar samples post-heating at 300 °C. As illustrated in Figure 2, the peak observed near 3400 cm^−1^ (wavenumber) corresponded to the stretching vibrations of -OH groups present in phenols, alcohols, acids, and similar compounds. The symmetric or asymmetric vibration peaks of -CH₂ and -CH₃ groups in aliphatic hydrocarbons can be observed near 2940 cm^−1^, although the absorption peaks in this region for biochar were relatively weak. This suggests that a significant portion of -CH₂ and -CH₃ groups undergo conversion into volatile substances or become fixed carbon during the carbonization process [21]. The peak near 1610 cm^−1^ was attributed to the stretching vibration of the C=C bonds in aromatic structures. Among the five biochar samples, MLB-300 exhibited a more pronounced peak at around 1600 cm^−1^ compared to the other four biochar samples. This can be attributed to the higher lignin content in mulberry leaves, resulting in stronger vibrations of the C=C bonds [22]. Additionally, stretching vibration peaks of alcohol groups can be observed around 1100 cm^−1^. 

### 2.2. Adsorption Characteristics

#### 2.2.1. Adsorption Kinetics

Adsorption kinetics plays a crucial role in understanding the specific adsorption process and the rate at which adsorbents interact with adsorbates, thereby directly influencing the adsorption effectiveness. Studying adsorption kinetics not only helps elucidate the mechanism of adsorption reactions but also aids in predicting the migration and destination behavior of heavy metal ions.

**Figure 3:**
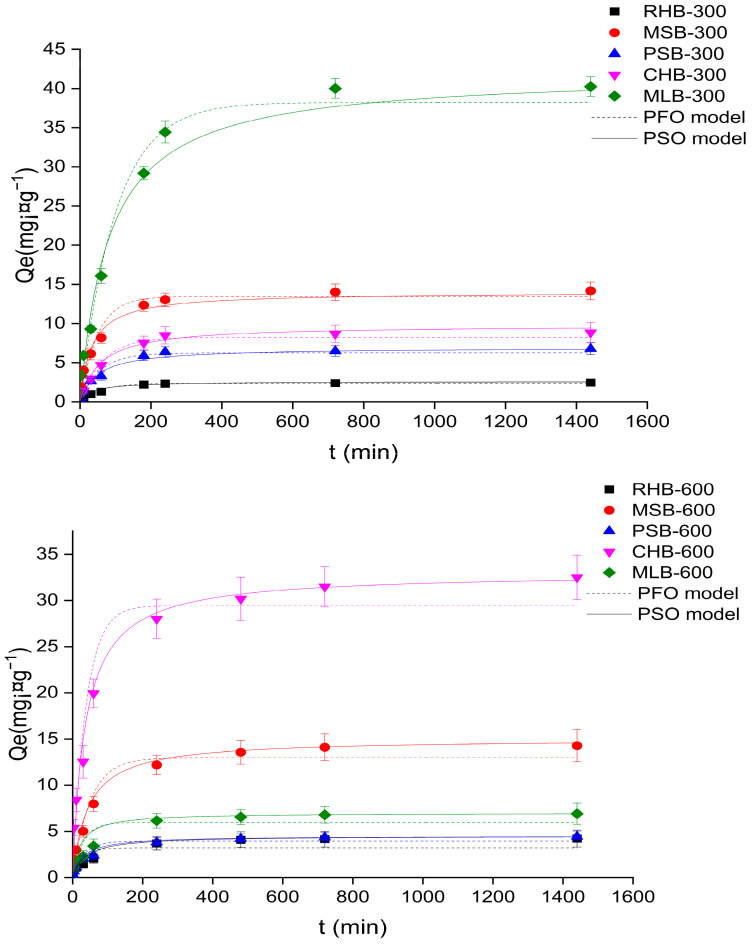
The adsorption data of Cd(II) fitted with pseudo-first-order kinetic and pseudo-second-order kinetic models.

Figure 3 illustrates the kinetic fitting diagram of Cd(II) adsorption by various biochars. The adsorption process can be divided into three stages: rapid adsorption, slow adsorption, and dynamic equilibrium. Initially, during the early stages of the adsorption reaction, the Cd(II) adsorption capacity of biochar increased with time. After 90 min of adsorption, the adsorption reached a state of equilibrium, indicating that biochar exhibited a relatively fast adsorption rate for Cd(II). In the initial stage, the adsorption amount increased rapidly due to the abundance of adsorption sites on the biochar, predominantly on its outer surface. As the adsorption process progressed, the adsorption sites on the biochar approached saturation, resulting in a slower adsorption rate until reaching adsorption equilibrium. Among the biochar samples prepared at the same temperature, MLB-300 demonstrated the highest adsorption capacity for Cd(II), reaching up to 38.23 mg·gcm^−1^, significantly surpassing the other four biochars.

The adsorption process was analyzed using the pseudo-first-order and pseudo-second-order kinetic models, and the results were presented in Table S2, which included the corresponding kinetic parameters. It was evident from the table that both models exhibited a good fit to the adsorption process (R^2^ > 0.95). However, the pseudo-second-order model showed a higher fitting coefficient and a closer agreement between the theoretical and actual adsorption values. This indicated that the pseudo-second-order kinetic model was more suitable for describing the adsorption of Cd(II) by biochar. It suggests that chemisorption, including processes like precipitation and complexation, predominantly contributes to the adsorption of Cd(II) by biochar.

#### 2.2.2. Sorption Isotherms

**Figure 4:**
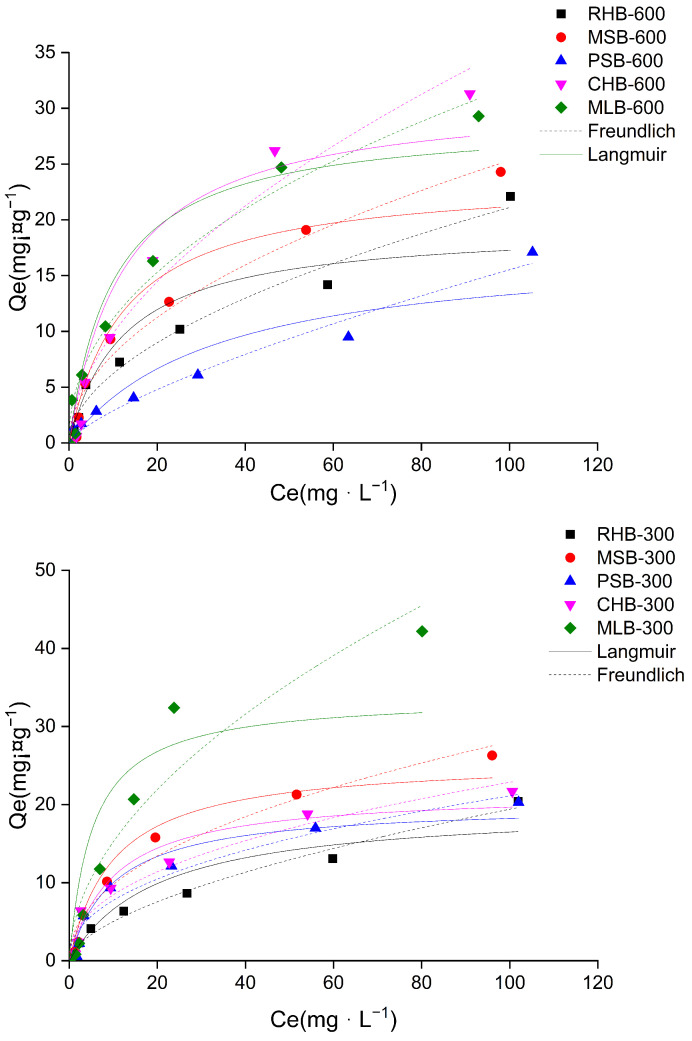
The adsorption data of Cd(II) fitted with the Langmuir model and the Freundlich model.

The isothermal adsorption curve provides valuable information about the maximum adsorption capacity of the adsorbent for heavy metals, offering insights into the adsorption behavior of heavy metals on the biochar surface. As depicted in Figure 4, an increase in the initial concentration of the Cd(II) solution led to a rapid enhancement in the adsorption extent of biochar for Cd(II). Subsequently, the adsorption process transitions from an initial fast stage to a slower stage until it eventually reaches equilibrium. At low concentrations of the Cd(II) solution, the biochar surface possessed an abundance of adsorption sites, resulting in a rapid adsorption rate. However, as the initial concentration of metal ions rose, the available adsorption sites became occupied, ultimately reaching saturation (equilibrium).

Among the biochars prepared at the same pyrolysis temperature, MLB-300 exhibited superior adsorption effectiveness with a maximum adsorption capacity of 42.2 mg·g^−1^. However, biochars derived from the same biomass at different temperatures demonstrated a certain promotion effect on the adsorption of Cd(II) due to the increased temperature during the pyrolysis process. For instance, the maximum adsorption capacity of CHB-600 was 31.3 mg·g^−1^, surpassing that of CHB-300, which was 21.7 mg·g^−1^, except for both MLB and RHB.

To further elucidate the adsorption mechanism of Cd(II) on the biochar surface, the Langmuir equation and Freundlich equation were employed to fit the isothermal adsorption data of Cd(II) (referring to Table 2). The correlation coefficients were all above 0.880, indicating a good fit. Among RHB, MSM-600, PSM-600, CHB-600, and MLB-600, the Freundlich equation provided a better fit than the Langmuir equation, suggesting that the adsorption mechanism of these biochars for Cd(II) tended to involve multilayer adsorption on the surface of a heterogeneous medium. However, the biochar prepared through pyrolysis at 300 °C exhibited a preference for monolayer adsorption on the surface of a homogeneous medium.

#### 2.2.3. Effect of the pH on Cd(II) Adsorption

The pH value of the heavy metal solution is a crucial factor influencing the adsorption behavior of biochar. It has the ability to alter the surface charge of biochar and the morphology of heavy metals. The impact of solution pH on adsorption primarily occurs by modifying the charges of the adsorbent and adsorbate, subsequently influencing the electrostatic interaction between them. In this study, the adsorption of Cd(II) was investigated under varying solution pH values ranging from 4 to 8.

As depicted in Figure S1, an increase in pH led to an elevation in the adsorption capacity of MSB for Cd(II) at 300 °C. Significantly higher amounts of Cd(II) were adsorbed by the biochar at pH 4–5. However, when the pH reached 7 and 8, the adsorption capacity of biochar for Cd(II) remained relatively constant.

This phenomenon can be attributed to the abundance of H^+^ ions in low-pH solutions, leading to competition between H^+^ and Cd(II) for limited adsorption sites on the biochar surface. This competition increases the electrostatic repulsion on the biochar surface, which hinders the adsorption of Cd(II) ions. As the solution pH increases, the concentration of H^+^ ions decreases, diminishing the competitive advantage and allowing hydrolysis and precipitation processes to enhance the electrostatic attraction towards Cd(II). Consequently, more Cd(II) ions are adsorbed by the biochar.

Furthermore, the increase in solution pH promotes the deprotonation of carboxyl and phenolic hydroxyl groups on the biochar surface, resulting in the formation of -O- and -COO- groups. This exposes additional adsorption sites, thereby increasing the adsorption capacity for Cd(II) ions. However, once the pH value reaches a certain threshold, the adsorption capacity of the biochar for Cd(II) ions may stabilize or even decrease. This could be attributed to the presence of Cd (OH)^+^ and Cd (OH)_2_ species in the solution, which can affect the adsorption capacity of the biochar for Cd(II) ions.

#### 2.2.4. Effect of Ionic Strength on Cd(II) Adsorption

The presence of oxygen-containing compounds, such as nitrate and phosphate, in surface and aqueous solutions often coexists with heavy metal ions. To investigate the impact of anions on the adsorption equilibrium of Cd(II) on biochar, adsorption experiments were conducted at different concentrations of NO_3_^−^ ions (0.1 M, 0.01 M, and 0.001 M). As depicted in Figure S2, the adsorption capacity of biochar for Cd(II) tended to decrease with increasing NO_3_^−^ concentration. Several factors contribute to this outcome. Firstly, it is commonly understood that the addition of electrolytes reduces the thickness of the double electric layer and weakens the electrostatic interaction between the adsorbent and the adsorbate. At high ionic strength, charge-balancing ions surround the adsorption sites with opposite charges, partially neutralizing the charges and diminishing the electrostatic interaction between the adsorption sites and the adsorbents. Consequently, the adsorption of heavy metals by biochar is reduced.

Secondly, in the case of outer surface complex (OSSC) formation, there is an ion exchange competition between the added electrolyte ions and the adsorbed ions, leading to the inhibition of adsorption. As the ionic strength increases, the competition intensifies, resulting in a decrease in adsorption. Overall, the observed decrease in adsorption with increasing ionic strength can be attributed to the compression of the double electric layer and weakened electrostatic interactions, as well as the ion exchange competition between the electrolyte ions and the adsorbed ions on the biochar surface.

#### 2.2.5. FTIR Spectra Prior- and Post-Cd(II) Adsorption

FTIR spectra of MLB-300 before and after Cd(II) adsorption are presented in Figure S3. The wave number of the vibration peak corresponding to oxygen-containing functional groups remained unchanged, suggesting that the types of functional groups on the surface of MLB-300 remained consistent. However, there were differences in transmittance and peak area before and after adsorption. Lower transmittance in the infrared spectrum indicates a higher impurity content and a greater presence of light-absorbing substances in the measured object. A larger peak area indicates a greater abundance of functional groups. Notably, the vibration of biochar at 1030 cm^−1^ before adsorption shifted to approximately 1095 cm^−1^ in the carbon after adsorption, indicating the involvement of -CO and -OH groups in the Cd(II) adsorption reaction.

Uchimiya et al. [23] found that the interaction between heavy metal ions and electrons in the carbon-carbon double bond effectively removes Cd(II) from the solution. The π-system of cyclic aromatic hydrocarbons acts as a π-donor, and the electron-donating capacity of the π-donor increases with the number of associated rings. Xie et al. [24] pointed out that heterocyclic compounds act as weak cation-π binding agents and readily form Cd-π complexes. Based on the FTIR spectra before and after adsorption, a peak indicating the formation of an aromatic structure with -Ch was observed at 874–796 cm^−1^ prior to adsorption. After Cd(II) adsorption, the intensity of the peak near 874–796 cm^−1^ weakened, suggesting the presence of Cd-π complexes. The stretching vibrations of aromatic C=C and C=O bonds occurred around 1641 cm^−1^. Following adsorption, the peak position shifted and the intensity increased, indicating the formation of a stable structure with lower energy for the original π aromatic system. Yu et al. [25] reported an increase in Cd(II) adsorption capacity on biochar from 47.04 mg·g^−1^ to 197.84 mg·g^−1^, primarily attributed to the enhancement of the Cd(II)-π electron bonding mechanism. 

#### 2.2.6. XPS Spectra Prior- and Post-Cd(II) Adsorption

**Figure 5:**
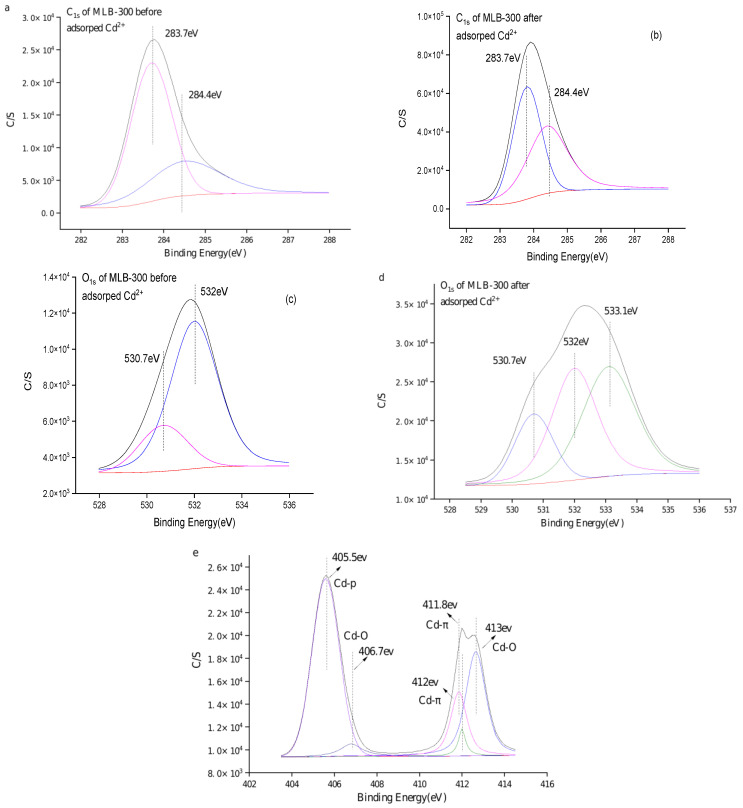
XPS spectra of mulberry biochar (treated at 300 °C) before and after Cd(II) adsorption. (**a**,**b**) C1s XPS spectra of MLB-300 before and after Cd(II) adsorption, respectively. (**c**,**d**) O1s XPS spectra of MLB-300 before and after Cd(II) adsorption, respectively. (**e**) Cd 2p XPS spectra of MLB-300 after Cd(II) adsorption.

Based on the XPS spectra of surface elements on biochar before adsorption (Figure 5), the elemental composition primarily consisted of carbon (C) and oxygen (O) elements, with binding energies of approximately 284 eV and 532 eV, respectively. The C1s peaks before adsorption can be fitted into two main chemical states of carbon (Figure 5a). These states corresponded to C-H, C-C, C=C, and C-O, with binding energies of 283.7 eV and 285.2 eV, respectively. The relative proportions of these states were as follows: C-H, C-C, and C=C account for 86.8%, while C-O accounts for 13.2%. The fitting of O1s peaks before adsorption (Figure 5c) revealed three main chemical states with corresponding binding energies of 530.7 eV, 531.5 eV, and 532.8 eV. These states corresponded to -OH, C-O, and C=O, accounting for approximately 28.0%, 5%, and 67.0%, respectively. Following adsorption, there was a change in the valence state of oxygen (Figure 5b). The percentages of COO-, C-O, and C=O increase to approximately 24.09%, 20.98%, and 54.93%, respectively. The decrease in C-O and C=O content indicated that these functional groups were primarily involved in surface complexation during the adsorption process, which was consistent with the findings of Zhang et al. [23]. Additionally, after Cd(II) adsorption, there was an increase in binding energy for C-O-C, C-OH, and C=O groups, suggesting that the oxygen atom acts as an electron donor for Cd(II). The electron density of the oxygen atom in these groups decreases, indicating that adsorption may occur through the formation of hydroxy-Cd and carboxy-Cd complexes. This aligns with the research conducted by Wang et al. [26]. The chemical state of carbon after adsorption (Figure 5d) remained unchanged; however, there was a shift in the proportion of functional groups. The percentages of C-H, C-C, and C=C increased to approximately 95.9%, while the proportion of C-O decreased to about 4.1%. This decrease in C-O content suggests that C-O functional groups were involved in the adsorption of Cd(II). Prior to adsorption, no Cd(II) was present on the biochar surface, as depicted in Figure 5e. After adsorption, the presence of Cd(II) was detected, and peak value fitting revealed two main states: Cd-O and Cd-π. The binding energies of Cd-O were measured at 406.7 eV and 413 eV, while the binding energies of Cd-π were 405.5 eV and 412 eV. The proportions of Cd-O and Cd-π were approximately 31.6% and 68.4%, respectively. These findings indicated that the Cd-π interaction was the primary mechanism for Cd(II) adsorption, which was consistent with the results obtained from Fourier infrared spectroscopy. In a study by Zhang et al. [27], it was demonstrated that Cd(II) adsorption predominantly occurred through coordination with π electrons, accounting for 54.1–82.6% of the total adsorption capacity, supporting the findings of this study.

#### 2.2.7. Correlation Analysis

Analyzing the impact of lignin and cellulose content in different biomasses on the adsorption performance of biochar is crucial. Understanding the relationship between lignin, cellulose, and adsorption capacity can aid in the targeted selection of biochar with excellent adsorption properties for heavy metals. This targeted approach can enhance adsorption efficiency and material selection. Biomass primarily consists of cellulose, hemicellulose, and lignin. Considering the thermal decomposition reactions of these three main chemical components, hemicellulose is the most unstable and decomposes in the temperature range of 225–325 °C. Cellulose decomposes at a higher temperature of 325–375 °C, while lignin gradually decomposes over a broader temperature range of 250–500 °C. The thermal decomposition of hemicellulose and cellulose results in many volatile products, while lignin mainly forms charcoal. During the carbonization temperature of 300–500 °C, intermediate products are formed from the polymerization of cellulose and lignin after they decompose. At 300 °C, some lignin remains. As the carbonization temperature increases, lignin decomposes gradually. During the pyrolysis process, cellulose and hemicellulose are first depolymerized into oligosaccharides, and then the glycosidic bonds are broken, producing D-pyran glucose. Through dehydration, decarboxylation, aromatization, and intramolecular condensation, solid carbon is formed. On the other hand, lignin mainly undergoes pyrolysis through free radical reactions. These free radicals are transferred to other substances, and when two free radicals collide, stable solid carbon compounds are formed. However, when the temperature is too high, the functional groups on the surface of biochar decrease, leading to a reduction in its adsorption capacity (temperature at 600 °C).

To investigate this relationship, linear regression analysis was performed using SPSS software 17.0 on lignin, cellulose, and adsorption capacity at 300 °C. The results are presented in Table S3. With a confidence interval of 95%, the linear regression analysis revealed a significant relationship between lignin and adsorption capacity, as indicated by a *p*-value of 0.034. This implied that the lignin content influenced the adsorption of Cd(II) by biochar derived from biomass. On the other hand, the *p*-value for the linear regression between cellulose and adsorption capacity was 0.064, suggesting that the relationship between cellulose and adsorption capacity is not statistically significant. The next step involved performing stepwise linear regression analysis using SPSS software on lignin, lignin plus cellulose, and adsorption capacity at 300 °C. The results are presented in Table S4. Initially, a linear regression analysis was conducted between lignin and adsorption capacity (300 °C). Subsequently, a linear regression analysis was performed on lignin plus cellulose and adsorption capacity (300 °C), and the outcomes are displayed in Table S4. By considering the results from both the single linear regression analysis and the stepwise linear regression analysis, it is evident that the lignin content in biomass significantly influences the adsorption capacity of Cd(II) after biochar preparation, while the presence of cellulose contributes to optimizing the model.

## 3. Materials and Methods

### 3.1. Chemical and Materials

This study examined the influence of both pyrolysis temperature and the type of biomass raw material on Cd adsorption. The experiment utilized five different biological feedstocks, each derived from agricultural residues (specifically, rice husk from Hunan province, maize straw from Tianjin city, peanut shell from Henan province, cottonseed shell velvet from Shandong province, and mulberry leaf from Zhejiang province). The raw materials were dried at 105 °C, ground using a grinder, and then pyrolyzed in an oxygen-resistant Muffle furnace with a heating rate of 10 °C/min and a nitrogen ventilation rate of 6 L/min. The pyrolysis temperatures were 300 °C and 600 °C, respectively, with a holding time of 2 h. After cooling to room temperature, the resulting biochar was screened using a 10 μm sieve, washed with deionized water, dried at 70 °C for 12 h, and then sealed in bags for later use. The biochar produced from rice husk, maize straw, peanut hull, cottonseed hull, and mulberry leaf at 300 °C was designated as RHB-300, MSB-300, PSB-300, CHB-300, and MLB-300, respectively, while the biochar produced at 600 °C was labeled RHB-600, MSB-600, PSB-600, CHB-600, and MLB-600.

The lignin and cellulose content of the five different biomass sources used in the experiment are summarized in Table S1. For the adsorption tests, cadmium chloride tetrahydrate (CdCl_2_·4H_2_O) and sodium nitrate (NaNO_3_) were utilized as reagents, both from Xilong Science Co. LTD. CdCl_2_·4H_2_O was prepared as a concentrated stock solution of 1000 mg·mL^−1^, while the NaNO_3_ solution was prepared at various concentrations. 

### 3.2. Adsorption Experiments

Kinetic adsorption tests were conducted by weighing 0.25 g of biochar and placing it in a 250 mL beaker. Next, 250 mL of cadmium nitrate tetrahydrate solution with a concentration of Cd(II) of 112.4 mg·L^−1^ was added to the beaker, and then the mixture was placed on a magnetic agitator at a constant temperature of 25 °C. Samples were taken at various time intervals (1, 3, 10, 30, 60, 120, 180, 300, 480, 720, and 1440 min), and 0.5 mL of each sample was extracted using a syringe with a 0.22 μm filter membrane and placed in a centrifugal tube. The concentration of Cd(II) in the filtrate was then determined using an atomic absorption spectrophotometer. The adsorption capacity of each biochar to Cd(II) was calculated based on the difference in Cd(II) concentration before and after reaching adsorption equilibrium, and the removal rate of Cd(II) was subsequently calculated.

(1) $$q_{e}=\left(\frac{C0-\mathrm{Ce}}{m}\right)V$$

In Equation (1), Where *C*_0_ and *C*_e_ are the initial and equilibrium concentrations (mg·L^−1^) of Cd(II). *m* is biochar mass (g), V is solution volume (L);

(2) $$Q_{t}=Q_{e}\left(1-e^{-k_{1}\cdot t}\right)$$

(3) $$Q_{t}=\frac{k_{2}\cdot Q_{e}^{2}\cdot t}{1+k_{2}\cdot Q_{e}\cdot t}$$

In Equations (2) and (3), where Q_t_ is the adsorption capacity (mg·g^−1^) of Cd(II) at time t; Q_e_ is the equilibrium adsorption capacity (mg·g^−1^); and K_1_ (h^−1^) is the constant of the pseudo-first order model equation. K_2_ (g·mg^−1^·h^−1^) is the adsorption rate constant of the pseudo-second-order kinetics model equation.

To conduct the isothermal adsorption experiment, 0.02 g of various biochar samples was placed in brown glass bottles, and 20 mL of cadmium solutions with different initial concentrations (0.88, 1.75, 3.5, 7, 14, 28.1, 56.2, and 112.4 mg·L^−1^) were added to each bottle. The mixture was then oscillated at 120 rpm at a constant temperature of 25 °C for 24 h. The suspension was filtered using Whatman filter paper, and the filtrate was collected and diluted at a certain multiple. The concentration of Cd(II) in the filtrate was measured using an atomic absorption spectrophotometer, and the adsorption capacity of each biochar sample for Cd(II) was calculated based on the difference in Cd(II) concentration in the initial and final solutions. The adsorption isotherms were fitted using the Langmuir and Freundlich models, where the Langmuir model includes the maximum adsorption capacity of the biochar and the Langmuir constant related to the energy of adsorption, while the Freundlich model includes the Freundlich constant and exponent indicating the adsorption capacity, intensity, and surface heterogeneity of the biochar for Cd(II).

Langmuir model:

(4) $$Q_{m}=\frac{Q_{m}\cdot K_{L}\cdot C_{e}}{1+K_{L}\cdot C_{e}}$$

Freundlich model:

(5) $$Q_{t}=K_{F}\cdot C_{e}^{N}$$

where Q_m_ is the maximum saturated adsorption capacity (mg·g^−1^). C_e_ is the equilibrium concentration of adsorbent in solution (mg·L^−1^). K_L_ (L·mg^−1^) and K_F_ (mg·g^−1^) were the adsorption coefficients of the Langmuir model and the Freundlich model, respectively. N is the Freundlich constant (Equations (4) and (5)).

To examine the impact of pH and ionic strength on the adsorption of Cd(II), 0.02 g of different biochar samples was added to brown glass bottles containing 20 mL of Cd(II) solution with varying initial concentrations (0.88, 1.75, 3.5, 7, 14, 28.1, 56.2, 112.4 mg·L^−1^). The pH of the solution was adjusted to a range of 4–7 using HCl and NaOH, and the solution was agitated for 24 h at 25 °C before filtration. The concentration of Cd(II) in the filtrate was measured. Moreover, the effect of different ionic strengths was studied by conducting isothermal adsorption experiments using 0.1 mol·L^−1^, 0.01 mol·L^−1^, and 0.001 mol·L^−1^ NaNO_3_ solutions. 

### 3.3. Characterization and Analysis

The surface morphology of biochar was scanned by an electron microscope (SEM). X-ray photoelectron spectroscopy (XPS) was used to measure and identify the functional groups on the biochar surface, and the element composition on the sample surface was analyzed. Fourier transform infrared spectroscopy (FTIR) was used to identify and display the surface functional groups of biochar. Based on nitrogen adsorption and desorption, the specific surface area and pore size of biochar were determined by a BET-specific surface area analyzer. The contents of carbon (C), hydrogen (H), nitrogen (N), and sulfur (S) in biochar were determined by elemental analysis.

### 3.4. Statistical Analyses

Statistical analyses were performed using SPSS v.17.0 for Windows software (SPSS Inc., Chicago, IL, USA). Means were compared by one-way analysis of variance and Duncan’s multiple range tests at the 5% level (significant difference *p* < 0.05).

## 4. Conclusions

Gaining a comprehensive understanding of biochar properties and effectively controlling its ability to adsorb harmful heavy metals is crucial for developing cost-effective and engineered biochar composites with high adsorption capabilities for soil remediation projects. The surface properties of biochar, prepared from various sources such as rice husk, straw, peanut shell, cottonseed shell, and mulberry leaf, directly influence the adsorption mechanism, ultimately determining the adsorption capacity of biochar at both 300 °C and 600 °C. Among the biochar samples investigated in this study, mulberry leaf biochar prepared at 300 °C exhibited the most effective adsorption performance for Cd(II), achieving a capacity of 42.20 mg·g^−1^. However, biochar prepared at 600 °C displayed poor adsorption efficiency for heavy metals, primarily due to the degradation of functional groups at high temperatures.

## Figures and Tables

**Table 1:** Physiochemical properties of the biochar.

	Bulk Element Composition (%)				Ash Content (%)	S_BET_ (m^2^g^−1^)	V_TPV_ (cm^3^g^−1^)	Pore Width (nm)
	C	H	O	N				
RHB-300	54.51	3.79	40.14	1.56	23.55	19.37	0.009	1.84
RHB-600	59.00	1.79	38.02	1.19	32.58	268.81	0.134	2.00
MSB-300	61.96	4.19	31.14	2.71	13.55	13.50	0.007	2.10
MSB-600	70.79	2.04	24.95	2.22	17.59	229.09	0.126	2.20
PSB-300	64.29	4.11	30.48	1.12	7.7	1.59	0.004	10.85
PSB-600	76.97	2.28	19.76	0.99	12.05	292.31	0.142	1.94
CHB-300	57.97	3.84	36.57	1.62	17.44	3.26	0.008	10.01
CHB-600	71.01	1.97	25.95	1.07	21.03	141.92	0.070	1.97
MLB-300	54.64	4.84	35.25	5.27	19.05	39.93	0.022	2.17
MLB-600	53.26	1.83	47.27	4.16	32.35	26.46	0.029	4.43

**Table 2:** Adsorption isotherm parameters of Cd(II) adsorption on different biochar.

	Langmuir			Freundlich		
	Q_max_ (mg g^−1^)	K_1_	R^2^	K_f_	1/n	R^2^
RHB-300	19.79 ± 3.500	0.05	0.921	1.305	0.586	0.986
RHB-600	19.43 ± 2.144	0.08	0.899	1.835	0.531	0.975
MSB-300	25.87 ± 0.564	0.1	0.971	3.315	0.457	0.964
MSB-600	23.82 ± 0.562	0.08	0.954	2.482	0.504	0.971
PSB-300	20.05 ± 1.209	0.1	0.961	2.838	0.436	0.946
PSB-600	17.73 ± 1.249	0.03	0.887	0.537	0.732	0.972
CHB-300	21.64 ± 2.268	0.1	0.967	3.124	0.432	0.953
CHB-600	31.26 ± 0.903	0.08	0.936	2.727	0.557	0.955
MLB-300	33.86 ± 5.238	0.19	0.979	4.543	0.526	0.932
MLB-600	29.04 ± 0.729	0.1	0.956	3.891	0.457	0.970

## Data Availability

The original contributions presented in the study are included in the article (and Supplementary Material), further inquiries can be directed to the corresponding authors.

## Supplementary Materials

The following supporting information can be downloaded at: https://www.mdpi.com/article/10.3390/molecules29153545/s1.
