# Supplementary material for: Efficacy of Agricultural Residue-Derived Biochar for Tackling Cadmium Contamination in an Aqueous Solution
Source: Molecules. 2024 Jul 27;29(15):3545. doi: 10.3390/molecules29153545 (PMC11314051; doi:10.3390/molecules29153545)
Supplement: Supplementary file 1 [file molecules-29-03545-s001.zip › molecules-3104212-supplementary.pdf]

Supplementary information

S-Tables

Table S1. Content of lignin and cellulose and ratio between two components in different biomass sources

Biomass	lignin(%)	cellulose(%)	lignin:cellulose
Rice husk	24.6	43.8	1:2
Corn stover	13.1	49.0	1:4
Peanut shells	34.7	40.2	1:1
Cotton seed shell hair	14.0	70.0	1:5
Mulberry leaf	32.8	18.9	2:1

Table S2. Kinetic parameters of Cd(II) adsorption on different biochar

Adsorbents	Pseudo first-order			Pseudo second-order		
	Q_e (mg g^{-1})	K_1	R^2	Q_e (mg g^{-1})	K_2 (g mg^{-1} min^{-1})	R^2
RHB-300	2.358±0.062	0.018	0.954	2.596±0.051	0.011	0.965
RHB-600	4.073±0.076	0.017	0.945	4.528±0.054	0.006	0.971
MSB-300	13.464±0.009	0.017	0.981	14.000±0.096	0.002	0.987
MSB-600	14.018±0.045	0.010	0.964	15.146±0.001	0.001	0.991
PSB-300	6.253±0.142	0.016	0.947	6.917±0.067	0.003	0.980
PSB-600	3.962±0.122	0.024	0.954	4.557±0.046	0.007	0.976
CHB-300	8.247±0.083	0.016	0.953	9.800±0.075	0.002	0.966
CHB-600	31.866±0.372	0.010	0.990	33.184±0.244	0.008	0.997
MLB-300	38.235±1.189	0.010	0.962	42.000±0.786	0.003	0.975
MLB-600	6.800±0.025	0.013	0.964	7.053±0.049	0.006	0.977

Table S3. Linear regression model parameters of lignin, cellulose and adsorption capacity (300°C)

Model	R^2	F	*P*
Lignin	0.448	6.503	0.034
Cellulose	0.366	4.614	0.064

Table S4. Stepwise linear regression model of lignin, cellulose and adsorption capacity (300°C)

Model	R^2	F	*P*
Lignin	0.448	6.503	0.034
Lignin and cellulose	0.317	950.4	0.018

S-Figures

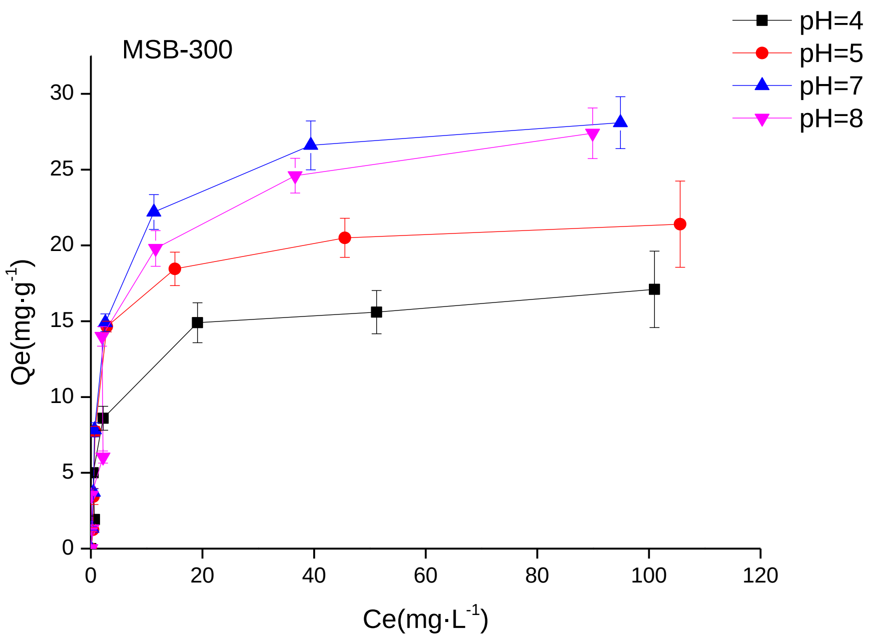

Figure S1. Adsorption isotherm of maize straw biochar (300 °C) at various pH levels

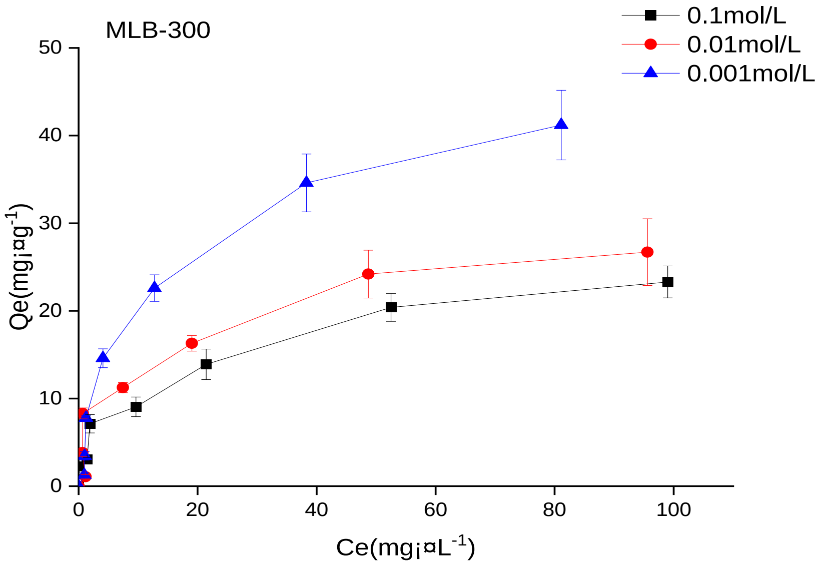

Figure S2. Adsorption isotherm of mulberry leaf biochar (300 °C) affected by various ionic strengths

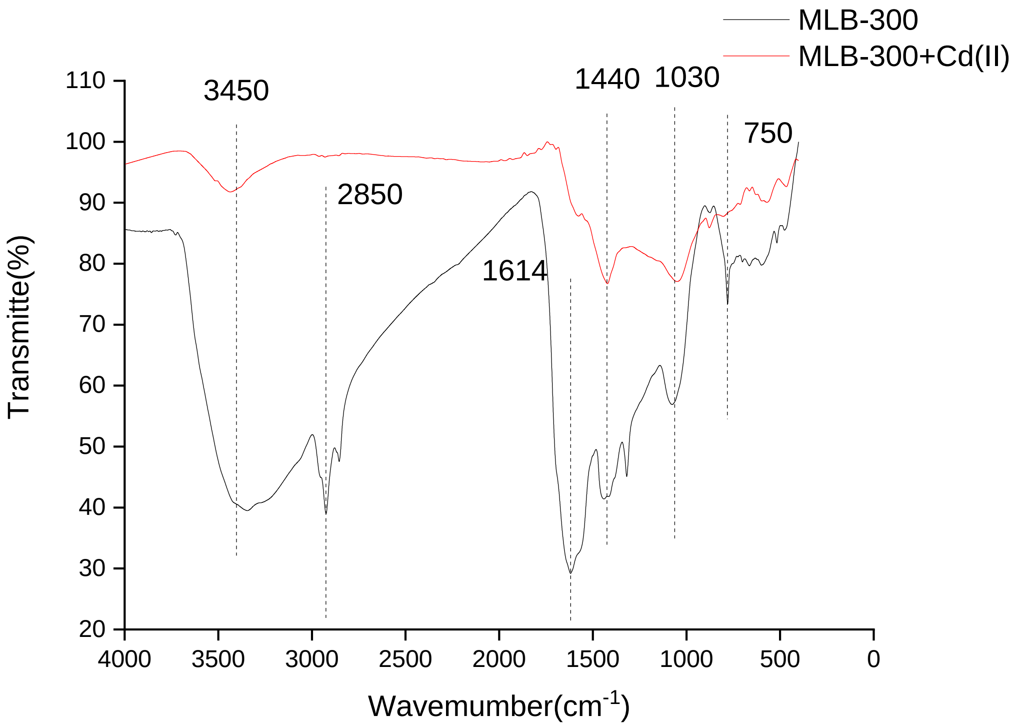

Figure S3. FTIR spectra of mulberry biochar (300 °C) before and after Cd(II) adsorption
